# Leadless Pacemaker Implantation in Fontan Patients with Multimodality Imaging: Tips and Tricks

**DOI:** 10.19102/icrm.2024.15082

**Published:** 2024-08-15

**Authors:** Srikant Das, Brian A. Boe, Joshua Saef, Kak-Chen Chan, Orhan Kilinc, Steven Bibevski, Todd S. Roth

**Affiliations:** 1 Adult Congenital Heart Disease Center, Memorial Cardiac and Vascular Institute, Joe DiMaggio Children’s Hospital, Hollywood, FL, USA; 2 Division of Pediatric Cardiology, Joe DiMaggio Children’s Hospital, Hollywood, FL, USA; 3 Division of Pediatric Cardiothoracic Surgery, The Pediatric Heart Institute, Joe DiMaggio Children’s Hospital, Hollywood, FL, USA

**Keywords:** Fontan, leadless pacemaker, Micra, transcatheter pacing

## Abstract

Current leadless pacemaker (LP) systems, which have been developed and used in patients with normal cardiac anatomy, are rare and technically even more challenging to implant in patients with congenital heart diseases, especially with univentricular physiology and Fontan palliation. We report two cases of percutaneous LP implantation in an adult and a child, respectively, highlighting the unconventional approaches, different challenges, and use of multimodality imaging in patients who underwent a Fontan operation.

## Introduction

**Figure 1:**
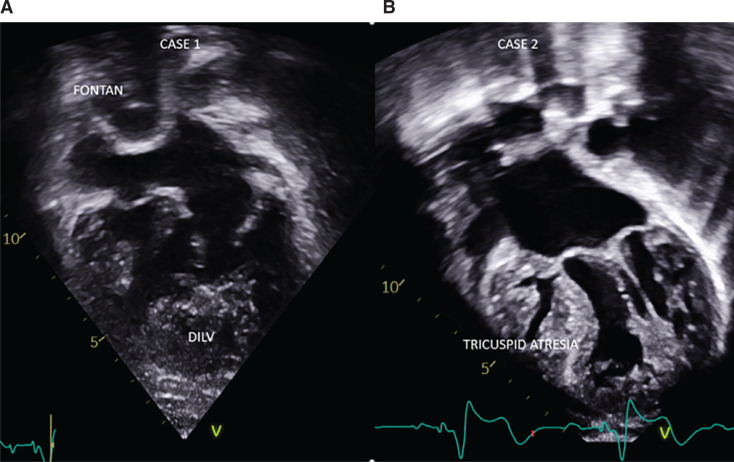
Echocardiographic views demonstrating single ventricular anatomy of the two Fontan cases. **A:** Case 1 with double inlet left ventricle (status post–lateral tunnel Fontan). **B:** Case 2 (right image) with tricuspid atresia and hypoplastic right ventricle in a patient with heterotaxy syndrome (left atrial isomerism, interrupted inferior vena cava with azygous continuation).

Transcatheter-delivered, fully encapsulated active fixation-based leadless pacemaker (LP) systems offer an excellent alternative for endocardial/epicardial approaches to address pacing needs in patients with structurally normal hearts.^1^ Patients with congenital heart disease are a unique group of patients with additional considerations secondary to a lack of venous access to cardiac chambers, complex intracardiac anatomy, higher risk of thromboembolic events, infection, and the need for atrioventricular (AV) synchrony. Optimal and safe techniques for the implantation of an LP system in patients with congenital heart disease and especially in those with univentricular physiology after Fontan completion have not yet been established, and reports are scarce.^2–5^ The Micra™ transcatheter pacing system (TPS) (Medtronic, Minneapolis, MN, USA) is typically implanted in the catheterization laboratory under fluoroscopic guidance. The device is mounted on its own dedicated 27-French (Fr) introducer sheath and advanced to the right ventricle (RV) percutaneously through the femoral vein with the aim of a septal position with fixation achieved by deployment of two of four nitinol tines. We report two cases of percutaneous LP implantation, in an adult (case 1) and a child (case 2), highlighting the unconventional approaches and different challenges in patients who underwent a Fontan operation **(Figure 1)**. Both patients had complete heart block with pre-existing epicardial pacemaker system implants and pacemaker dependence. Case 1 was a patient who had a double inlet left ventricle (DILV), status post–lateral tunnel type of Fontan palliative surgery, thus excluding direct access to cardiac chambers via the superior vena cava (SVC) and inferior vena cava (IVC). Case 2 was a 38-kg child who had tricuspid atresia and hypoplastic RV with heterotaxy syndrome (left atrial isomerism, interrupted IVC with azygous continuation). He was status post–extracardiac fenestrated Fontan procedure with a Kawashima operation, bilateral bidirectional Glenn anastomosis, and creation of the right pulmonary artery (RPA) to right atrium fenestration and hepatic veins to the RPA conduit. Thus, direct access to cardiac chambers was absent, making approaches to Micra™ implantation challenging and non-traditional.

## Case 1

**Figure 2:**
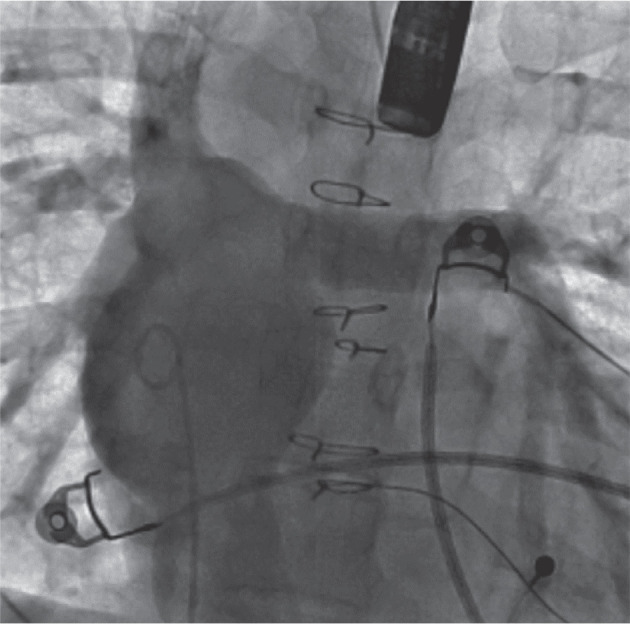
An angiogram (case 1) in the inferior vena cava demonstrates the typical appearance of a lateral tunnel Fontan without any fenestration and connections of the inferior and superior vena cavae with the pulmonary arteries.

A 32-year-old woman with univentricular physiology, DILV, and status post–lateral tunnel Fontan **(Figures 1 and 2)**, with complete AV block, presented with recurrent epicardial system infections caused by *Enterobacter cloacae* and pacemaker pocket dehiscence. She had multiple pocket revisions, prolonged antibiotic therapy, and wound vacuum application to abdominal sites. After discussing the possible options and associated procedural risks (redo sternotomy, revision of leads and pocket), we decided to proceed with LP implantation. Due to her univentricular physiology, a Micra™ AV (Model MC1VR01; Medtronic, Inc., Minneapolis, MN, USA) leadless system was chosen for implantation. This device offers detection of atrial contractions using the device’s built-in three-axis accelerometer to provide AV synchronous (VDD) pacing.^6^

### Implantation technique

**Figure 3:**
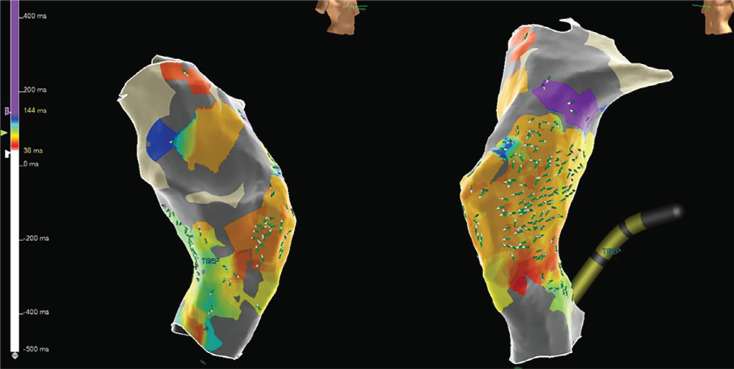
Electroanatomic and voltage mapping with activation vectors displays electrical activation of the Fontan chamber originating at the inferomedial aspect signifying anatomic and electrical contiguity in the region between the common atrium and surgically fashioned lateral tunnel/Fontan (case 1). This area was chosen as the site of puncture to enter the common atrium.

**Figure 4:**
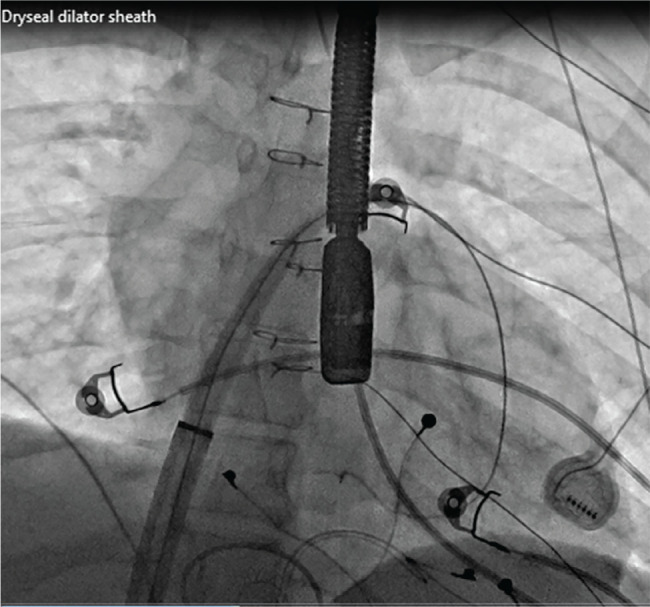
A 26-Fr DrySeal sheath and dilator assembly is advanced after transbaffle puncture into the common atrium with the wire tip in the DILV (case 1).

**Figure 5:**
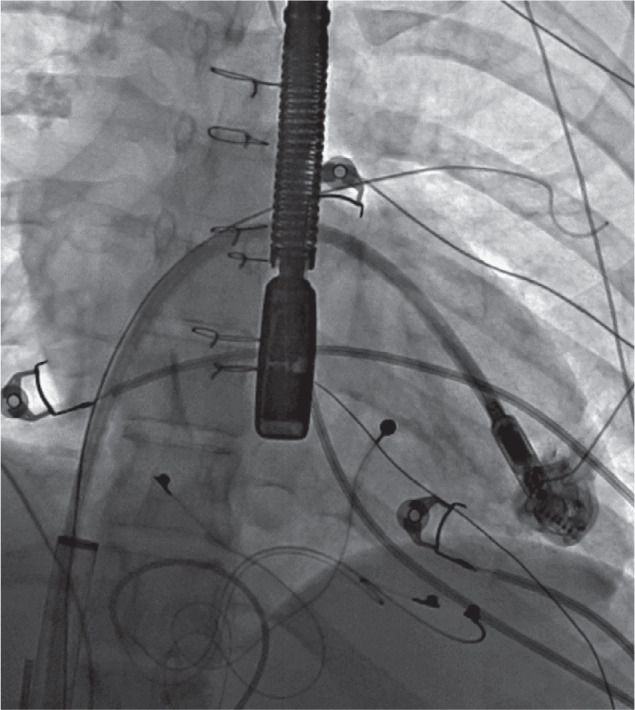
A fluoroscopy image showing the 26-Fr DrySeal sheath withdrawn into the Fontan and the Micra™ delivery catheter and the LP advanced into the DILV (case 1). A separate 0.035-in wire is noted in the LUPV advanced outside the Micra™ delivery catheter across the same puncture site through the DrySeal sheath.

**Figure 6:**
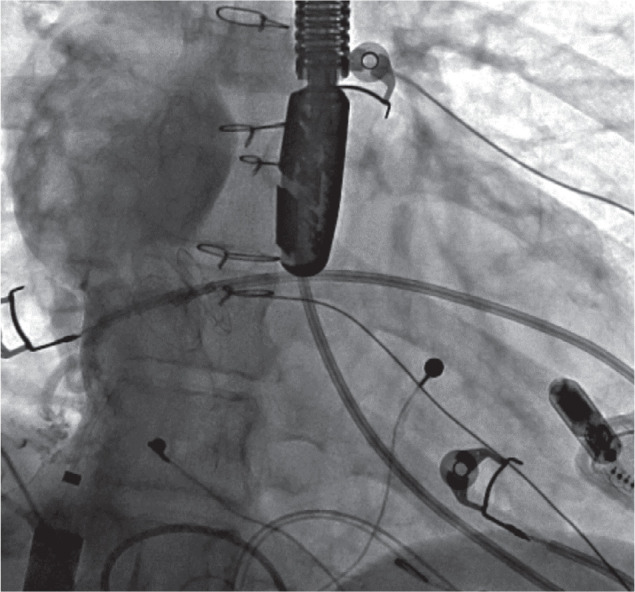
A cineangiogram view demonstrating a 20-mm GORE^®^ Cardioform Septal Occluder deployed at the site of puncture without any contrast flow in the common atrium (case 1). Also seen is the Micra™ leadless pacemaker in the double inlet left ventricle.

After the patient was placed under general endotracheal anesthesia and given intravenous antibiotics, an 8-Fr sheath was placed in the right femoral vein and a 10-Fr sheath was placed in the left femoral vein. An intracardiac echocardiography (ICE) catheter (ViewFlex™ Xtra ICE Catheter; Abbott, Chicago, IL, USA) was also inserted into the left femoral venous sheath and advanced to the Fontan. Imaging with transesophageal echocardiography (TEE), ICE, and angiography in the Fontan baffle showed no fenestration **(Figure 2)**. Electroanatomic mapping (EAM) in the Fontan baffle **(Figure 3)** was used to assess the relationship of the Fontan baffle with the pulmonary venous atrium to select an area of contiguity for a transbaffle puncture. A 64-pole Advisor™ HD Grid Mapping Catheter (Abbott) with three-dimensional (3D) EAM using the EnSite Precision™ Cardiac Mapping System (Abbott) was used to visualize the CARTO VIZIGO^®^ Bi-Directional Guiding Sheath (Biosense Webster, Diamond Bar, CA, USA) without fluoroscopy. The combined use of ICE and a steerable sheath visualized by a 3D mapping system provided procedural guidance with minimal radiation exposure, thus improving accuracy for the positioning of the transseptal needle (NRG™ RF Transseptal Needle; Baylis Medical, Montréal, QC, Canada). After selection of a site for radiofrequency (RF) puncture in the lower left side of the Fontan baffle, the RF transseptal needle was advanced from the Fontan to the common atrium. After crossing with the RF needle, the dilator and Vizigo sheath were advanced into the pulmonary venous atrium. The dilator was removed, and a 5-Fr MPA2 catheter was used to enter the left upper pulmonary vein (LUPV), where a 0.035-in Amplatzer™ Super Stiff wire (Boston Scientific, Marlborough, MA, USA) was positioned. The deflectable Vizigo sheath was exchanged for a 65-cm-long 26-Fr GORE^®^ DrySeal sheath (Gore Medical, Flagstaff, AZ, USA), which was advanced to the pulmonary venous atrium over a dilator **(Figure 4)**. The prepped Micra™ delivery catheter system was inserted into the DrySeal sheath next to the Amplatzer™ wire and advanced to the apex of the LV. Throughout this maneuver, the 0.035-in Amplatzer™ wire position was maintained across the fenestration in the LUPV. The DrySeal sheath was pulled back into the Fontan baffle to allow gentle navigation of the Micra™ delivery sheath across the left AV valve into the left ventricular apex **(Figure 5)**. The positioning of the implant was guided by fluoroscopic views, contrast injections through the DrySeal sheath, TEE, and ICE evaluation. The Micra™ TPS was deployed in the left ventricular apex, and several tug tests under fluoroscopy showed stable pacemaker positioning. The pacing and sensing parameters were adequate. The TEE confirmed that there was no entrapment of the valvular apparatus, and there was also no new-onset AV valve or neoaortic regurgitation. After removal of the Micra™ delivery catheter, there was significant right-to-left shunting with desaturation into the 70s. Using TEE evaluation, the Fontan fenestration was closed using a 20-mm GORE^®^ Cardioform Septal Occluder **(Figure 6)**. On follow-up, both the Micra™ TPS and GORE^®^ Cardioform Septal Occluder remained in a stable position with adequate pacing function characteristics. The patient underwent an uneventful removal of the infected epicardial pacemaker system.

## Case 2

A 15-year-old boy, weighing 38 kg, with univentricular physiology including tricuspid atresia and hypoplastic RV **(Figure 1)** with heterotaxy syndrome, left atrial isomerism, interrupted IVC, and bilateral SVCs presented with chest discomfort and chronic epicardial ventricular lead dysfunction with a high pacing threshold as well as intermittent loss of pacing capture and a pacemaker generator nearing depletion. He was status post–extracardiac fenestrated Fontan procedure with Kawashima operation, bilateral bidirectional Glenn anastomosis, and the creation of the RPA to right atrium fenestration and hepatic veins to the RPA conduit. Thus, direct access to cardiac chambers via the right and left SVCs, interrupted IVC, and hepatic veins was absent, making approaches to Micra™ implantation challenging and non-traditional. He had complete AV block with pacemaker dependence. Just 3 years before this presentation, he had undergone repeat sternotomy for the addition of a ventricular pacing lead due to lead failure and pacemaker generator replacement operation. That operation was difficult and complicated with prolonged, extensive surgical dissection for ventricular surface exposure. The lead body could not be directly secured to the ventricular surface and was stabilized using sutures to the shaft of the lead. He also had significant postoperative bleeding or chest tube drainage requiring a blood transfusion. After discussing the possible options and associated procedural risks (redo sternotomy, revision of leads and pocket, replacement of generator), we decided to proceed with percutaneous LP (Micra™ AV) implantation via a right internal jugular venous (RIJ) approach.

### Implantation technique

The procedure was performed under general endotracheal anesthesia. After positioning and draping the right side of the neck, percutaneous vascular access was obtained in the RIJ vein, and an 8-Fr sheath was placed. Heparin was administered, and the activated clotting time was monitored throughout the procedure.

**Figure 7:**
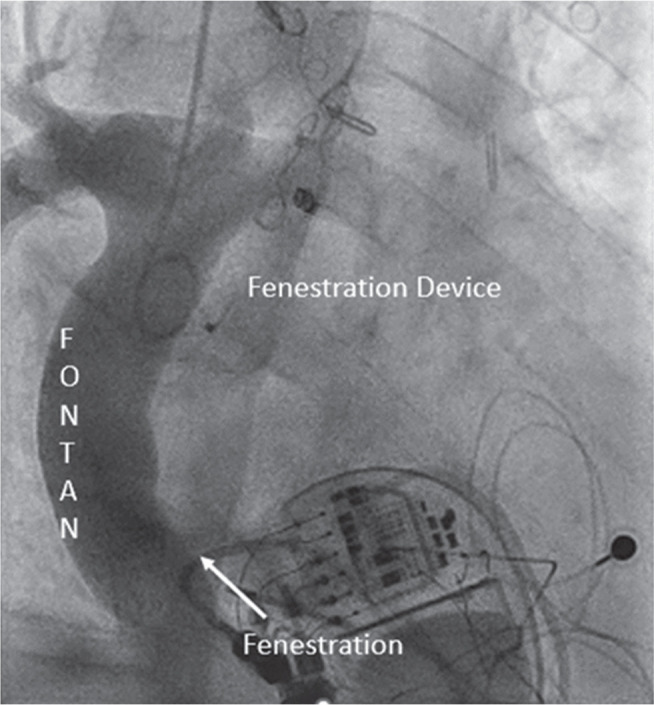
An angiogram (case 2) with a pigtail catheter in a Fontan baffle from a right internal jugular venous approach demonstrates a small fenestration inferiorly and leftward. A more superior fenestration site is noted with a septal occluder device in situ.

**Figure 8:**
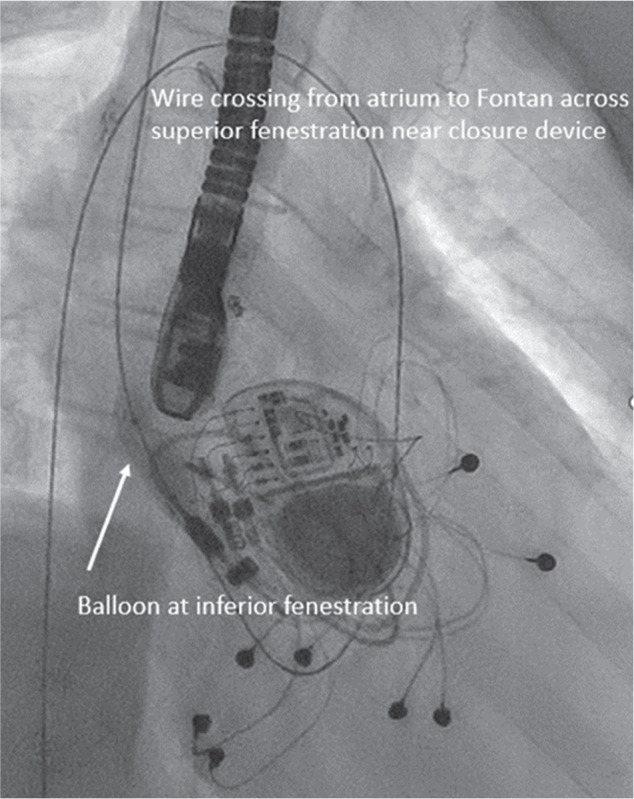
A fluoroscopic view (case 2) demonstrating the complex loop (right internal jugular vein → superior vena cava → Fontan → inferior fenestration → left atrium → superior fenestration → Fontan baffle) with a stiff 0.035-in Amplatz™ wire and balloon inflation at the inferior fenestration site.

**Figure 9:**
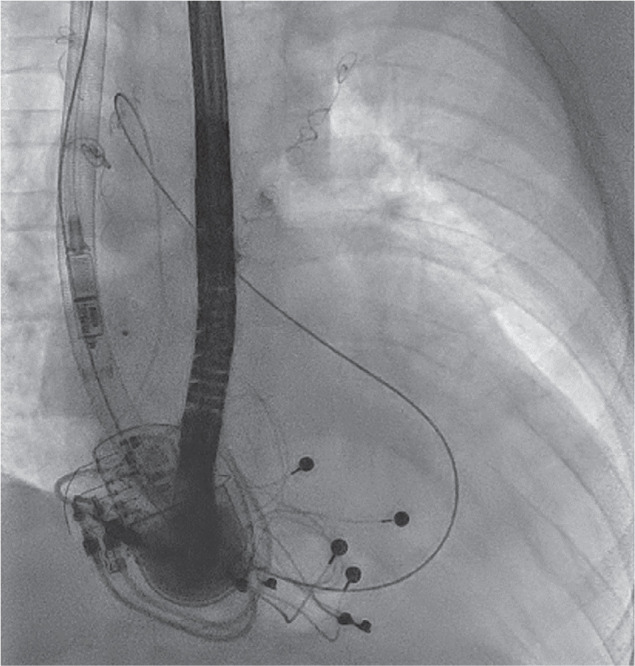
A fluoroscopic image (case 2) showing the Micra™ device advanced inside a GORE^®^ DrySeal Flex Introducer Sheath alongside the Amplatz™ wire, which is maintained across the fenestration in a complex loop (from right internal jugular vein → superior vena cava → Fontan → inferior fenestration → left atrium → superior fenestration → Fontan baffle).

A pigtail catheter was advanced, and angiograms were performed in the Fontan baffle to delineate anatomy and landmarks for transbaffle access via existing fenestration known by echocardiogram and prior angiogram **(Figure 7)**. There was a small fenestration with flow of contrast noted into the cardiac chamber at the inferior-left part of the Fontan baffle. Trans-Fontan baffle access was obtained by crossing with a 0.035-in GLIDEWIRE^®^ guidewire (Terumo Medical Corp., Somerset, NJ, USA) and a 4-Fr angled GLIDECATH^®^ catheter (Terumo Medical Corp., Somerset, NJ, USA) from the inferior fenestration. The wire and the catheter were advanced into the LV/single ventricle and across the aortic valve into the right innominate artery, but the Amplatz™ Super Stiff 0.035-in wire could not be exchanged and advanced due to the formation of a large loop in the atrium. The guidewire was then crossed back into the Fontan baffle from the left atrium (LA) via the superior fenestration into the RPA, creating a loop of SVC → Fontan → inferior fenestration → LA → superior fenestration → Fontan baffle. This loop was preserved by exchanging with an Amplatz™ Super Stiff wire and was used for the rest of the procedure. Angioplasty of the inferior fenestration was performed over this wire with a 10-mm OPTA^®^ PRO balloon (Cordis, Santa Clara, CA, USA) to facilitate advancement of the 26-Fr DrySeal sheath **(Figure 8)**. The RIJ access site was dilated with a Coons taper dilator (Cook Medical, Bloomington, IN, USA), and a 26-Fr, 65-cm GORE^®^ DrySeal Flex Introducer Sheath was introduced over the Amplatz™ wire for the delivery of the Micra™ device instead of the provided 27-Fr Micra™ introducer sheath. The Micra™ delivery catheter system was prepped and inserted into the DrySeal sheath by the side of the Amplatz wire and advanced to the apex of the LV under fluoroscopic and TEE guidance with gentle navigation **(Figure 9)**. Continuous assessment by TEE was performed to ensure crossing across the mitral valve without any entrapment or injury to the AV valve or other cardiac structures. The positioning and site selection of the implant were guided by multiple fluoroscopic views, small contrast injections, and TEE evaluation prior to applying pressure and deployment of the Micra™ device using the standard technique. The pacing and sensing parameters were excellent. The TEE confirmed that there was no entrapment of the valvular apparatus, and there was also no new-onset mitral valve or neoaortic regurgitation. After a brief discussion with the surgery and cardiac team, it was decided not to close the fenestration that was dilated at this juncture. The tether was cut and removed, which was followed by the removal of the delivery catheter and sheath. The device remained in a stable position. The patient tolerated the procedure well and was extubated in the catheterization lab.

Both patients were discharged from hospital and have been followed up with (case 1 for 5 months; case 2 for 1 month) in the outpatient setting without any thromboembolic complications or new concerns, and both reported a return to baseline activity of daily living.

## Discussion

Our report describes successful implantation of leadless Micra™ pacemakers with innovative approaches in challenging postoperative anatomy of two Fontan patients with complete heart block and high-risk limited alternatives. In case 1, a Micra™ AV TPS was successfully implanted via an IVC approach and a trans-Fontan baffle puncture in a patient with complex congenital heart disease, DILV, and lateral tunnel Fontan palliation. In case 2, a child weighing 38 kg with univentricular physiology involving tricuspid atresia with heterotaxy syndrome, left atrial isomerism, interrupted IVC, and bilateral SVCs, a Micra™ AV was implanted with an RIJ approach. This patient had a fenestrated Fontan procedure with a Kawashima operation, bilateral bidirectional Glenn anastomosis, and creation of the RPA to the right atrium fenestration and hepatic veins to the RPA conduit. In addition, a previous fenestration at a more favorable location was already closed by a septal occluder device. Thus, an RIJ venous approach with a trans-Fontan baffle access via an existing inferior fenestration was planned. In both cases, for Micra™ implantations, we used a 65-cm-long 26-Fr GORE^®^ DrySeal Flex Sheath with a hydrophilic coating that offers enhanced flexibility and kink resistance to cross the baffle instead of the 55-cm-long, 27-Fr Micra™ introducer sheath, which is prone to kinking. The use of the DrySeal sheath also allowed us to keep a wire in position outside of the Micra™ delivery catheter, and this wire was used to guide the delivery sheath for fenestration device closure in case 1 after Micra™ implantation, rather than recrossing the baffle. In case 2, due to the short length of the delivery sheath inside the body from the RIJ approach and a complex wire loop formation, it was helpful to perform balloon angioplasty over the stiff Amplatz wire at the fenestration and maintain the wire across the fenestration during Micra™ delivery.

In patients with various types of Fontan operations, postoperative anatomy can vary with areas of contiguity with the native atrium and discontinuous areas between the Fontan baffle and native atrium along its length. Thus, it is critically important to perform a trans-baffle puncture at a site of continuity prior to dilation of the puncture site. In case 1, the EAM system–guided voltage mapping, vector propagation, image integration, depiction of the chamber ultrasonic contour, and visualization of the sheath in conjunction with ICE helped identify the site of the trans-baffle puncture and reduce radiation exposure during the procedure. However, use of such a mapping system is not mandatory for all Fontan cases, especially those with pre-existing fenestration as demonstrated by the use of fluoroscopy and TEE only in case 2. Imaging guidance with TEE and/or ICE is crucial during transbaffle puncture and manipulation of the Micra™ delivery catheter through the fenestration and AV valve into the ventricular site without injury or impingement of the valvular apparatus.

LP systems, especially those that offer AV synchrony, are an attractive alternative to epicardial systems for the complex congenital heart disease population. The prevalence of LP-related infections is low due to the absence of a subdermal surgical pocket and pacemaker leads. There are increasingly innovative and feasible ways to deliver these devices in an intracardiac position, though it is imperative that risks and benefits be discussed by a multidisciplinary adult congenital heart disease team with procedural experience.^2–5^ The mechanical atrial sensing algorithm produced 60%–64% AV synchrony on follow-up 2–4 weeks later. Further programming assessment and adjustments were made at follow-up visits to optimize and increase AV synchrony.

Fontan palliation, which routes systemic venous blood directly to the pulmonary vascular bed with the lack of a subpulmonic pump, leads to significant circulatory challenges and complications due to chronic low cardiac output, high central venous pressure, and venous stasis. Thrombus formation and the risk of thromboembolic events are increased due to various factors, such as low-velocity non-laminar flow, prosthetic material, endothelial dysfunction, blind-ending pouches, atrial arrhythmias, and altered procoagulant and anticoagulant factors.^7,8^ Consequently, many, if not all, patients with a Fontan need antithrombotic prophylaxis, although regimens vary. The adult in case 1 was already on a non-vitamin K antagonist oral anticoagulant (NOAC) that was continued, and case 2 was started on a NOAC after Micra™ implantation. The child in case 2 was on aspirin only prior to the Micra™ implantation procedure. More cases and longer follow-up data are needed for an ideal anticoagulation strategy for an LP indwelling in a systemic ventricle.

## Conclusion

Transcatheter LP implantation in complex congenital heart disease can be aided by multimodality imaging as well as innovations in standard equipment and techniques during the implantation procedure.
